# The Early Effects of Esketamine on the Tumor Metastatic Microenvironment in Postoperative Lung Cancer Patients

**DOI:** 10.1111/crj.70108

**Published:** 2025-08-13

**Authors:** Yong Wang, Weijing Li, Li Jia, Junmei Shen, Chao Li, Huiqun Jia

**Affiliations:** ^1^ Department of Anesthesiology The Forth Hospital of Hebei Medical University Shijiazhuang Hebei Province China

**Keywords:** esketamine, lung cancer, MMP‐9, TNF‐α, tumor microenvironment metastasis, VEGF‐C

## Abstract

**Background:**

To investigate the early effect of esketamine on the tumor metastatic microenvironment in patients with lung cancer.

**Methods:**

Sixteen adults aged 45–80 years with the American Society of Anesthesiologists (ASA) 1 to 3 were randomly divided into the experimental group (group E) and the control group (group C). Group E received esketamine at 1 mg/kg during anesthesia induction and a continuous infusion of 0.5 mg/kg/h during the surgery. Group C was given the same amount of normal saline infusion. Patient‐controlled intravenous analgesia (PCIA) in group E was administered using dexmedetomidine (0.5 mg/kg) + esketamine (50 mg) + dexamethasone (5 mg). PCIA in group C was the same dose of dexmedetomidine and dexamethasone. Data were recorded at 14 points from admission to the third day after surgery (T_0–14_). Parameters recorded included hemodynamics, wake time, remifentanil dosage, and so on. At T_0_, T_10_, T_13_, and T_14_, TNF‐α, IL‐2, IL‐10, MMP‐9, and VEGF‐C were measured.

**Results:**

Compared with T_0_, the differences of tumor necrosis factor‐α (TNF‐α), interleukin‐2 (IL‐2), matrix metallopeptidase 9 (MMP‐9), and vascular endothelial growth factor‐C (VEGF‐C) in the two groups were statistically significant (*p* < 0.05). When compared to group C, VEGF‐C in group E was reduced at T_10_ and T_13_ (*p* < 0.05). For both groups, there were intragroup differences in the changes of MMP‐9 and VEGF‐C levels (*p* < 0.05). Compared to group C, on the postoperative, group E exhibited a lower change rate of TNF‐α and VEGF‐C (*p* < 0.05).

**Conclusion:**

Perioperative application of esketamine in patients with lung cancer provided significant sedative and analgesic effects and affected cytokines in the tumor microenvironment.

## Graphical abstract

The perioperative administration of esketamine in lung cancer patients prolonged the awakening time, reduced the dosage of remifentanil, and suppressed inflammatory responses.

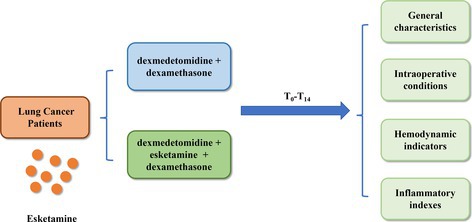

## 1 Introduction

With changes in the human societal landscape and advancements in tumor detection technology, the diagnostic rate of tumors is increasing [1, 2]. Surgical resection stands as one of the primary treatments for lung cancer, while tumor recurrence and metastasis are the foremost causes of mortality in such patients [3]. Tumor microenvironment metastasis plays a pivotal role in tumor recurrence and distant metastasis, significantly influencing long‐term patient prognosis. Tumor microenvironment metastasis refers to the specialized organ‐tissue environment formed before the migration of in situ tumors, which facilitates the creation of a microenvironment conducive to tumor cell dissemination [4]. In the tumor microenvironment of metastasis, the inflammatory response encompasses several pro‐tumor activities [5, 6, 7, 8] . During inflammation, the release of inflammatory and microenvironmental factors is promoted, resulting in the recruitment and activation of macrophages, neutrophils, and fibroblasts. These factors stimulate residual cancer cell survival, proliferation, and migration at the resection site, thus fostering angiogenesis and metastasis.

Surgical trauma can induce an inflammatory response, compromise the body's immune defense function, and trigger stress reactions [9]. Appropriate perioperative anesthesia management is an effective approach to reducing perioperative stress responses [10, 11]. However, different anesthesia methods and anesthetic agents can influence the release of inflammatory and microenvironmental factors, affecting the immune profile of cancer patients, thereby altering the tumor microenvironment [12, 13], and consequently impacting the treatment and prognosis of tumors [14, 15]. Therefore, perioperative anesthesia management indeed influences immune responses and the tumor microenvironment of surgical patients from various angles, exerting a certain influence on the long‐term prognosis of cancer patients. Clinical studies on tumors play an irreplaceable role in guiding scientifically rational choices for perioperative anesthesia management. This endeavor can contribute to reducing tumor recurrence and metastasis, thereby enhancing the long‐term prognosis of cancer patients [16, 17]. As a domestically produced new anesthetic, compared to ketamine, esketamine is the right‐handed enantiomer of ketamine and has a higher affinity for N‐methyl‐D‐aspartic acid (NMDA) receptors and μ‐opioid receptors, resulting in stronger analgesic and sedative effects. Research has indicated that combining propofol with esketamine for general anesthesia can lead to smoother hemodynamics, reduce surgical stress responses, exhibit notable anti‐inflammatory effects, and promote postoperative cognitive function recovery in patients [18]. For cancer patients, ketamine has been shown to exert antitumor effects by antagonizing NMDA receptors on cancer cells [19, 20], while simultaneously reducing vascular endothelial growth factor (VEGF) expression [21], thereby improving the tumor microenvironment to inhibit tumor recurrence and metastasis [22]. However, whether perioperative administration of esketamine provides similar or superior anti‐inflammatory, chronic pain development inhibition, immune regulatory protection, and its early effects on the tumor immune system, as well as its impact on the tumor microenvironment in terms of metastasis, remains to be studied in clinical settings.

This study focuses on patients undergoing thoracoscopic lung cancer resection surgery. Perioperative administration of esketamine is applied, with tumour necrosis factor‐α (TNF‐α), interleukin‐2 (IL‐2), IL‐10, matrix metallopeptidase 9 (MMP‐9), and vascular endothelial growth factor‐C (VEGF‐C) serum concentrations as the primary indicators, combined with secondary indicators such as hemodynamics. The aim is to assess the immunological aspects of esketamine in relation to tumors, evaluate its effects on the perioperative tumor microenvironment of lung cancer resection patients, and provide theoretical reference for optimizing anesthesia management during the perioperative period for cancer patients.

## 2 Materials and Methods

### 2.1 Materials

#### 2.1.1 Inclusion Criteria

Patients scheduled for thoracoscopic lung cancer surgery at the Fourth Hospital of Hebei Medical University (aged 45–65); Anesthesiologists Classification System (ASA) grades I and II; without history of mental illness; no known allergies to anesthesia drugs.

The study was conducted in accordance with ethical principles of the Declaration of Helsinki, and all patients provided written informed consent before entering the study.

#### 2.1.2 Exclusion Criteria

Cases with contraindications to alprazolam use and cases where ethical refusal is present; severe hypertension ([systolic blood pressure, SBP] ≥ 180 mmHg/[diastolic blood pressure, DBP] ≥ 110 mmHg); severe cardiovascular or cerebrovascular diseases (significant sequelae from stroke or cerebral hemorrhage, severe heart diseases); alcohol abuse.

#### 2.1.3 Removal Criteria

Experiments will be discontinued for cases with significant intraoperative bleeding (reaching or exceeding 800 mL within a short period or 20% of total circulating blood volume) or for cases with severely prolonged surgical duration (more than twice the average duration for similar surgeries in the country, ≥6 h).

### 2.2 Methods

#### 2.2.1 Study Population

A total of 16 patients undergoing elective thoracoscopic lung cancer surgery in our hospital from October 2022 to February 2023 were selected. They were randomly divided into the experimental group (group E, *n* = 8) and the control group (group C, *n* = 8). All the selected samples were non‐small cell lung cancer patients who had not received preoperative neoadjuvant treatment, and the tumor subtypes of the experimental group and the control group were adenocarcinoma and squamous cell carcinoma, and the ratio of adenocarcinoma/squamous cell carcinoma was 3/5.

#### 2.2.2 Anesthesia Protocol

Upon entering the operating room, a peripheral intravenous line was established in the upper limb for vascular access. Cardiac monitoring (electrocardiography [ECG], heart rate [HR], and oxygen saturation) was initiated. Under ultrasound guidance, radial artery cannulation was performed for invasive arterial pressure monitoring. The bispectral index (BIS) monitor was connected to assess anesthesia depth.

After triple verification, anesthesia induction was initiated. The experimental group (group E) received intravenous alprazolam at a dose of 1 mg/kg within 1 min prior to induction, followed by continuous infusion at a rate of 0.5 mg/kg/h until 20 min before the end of surgery. The control group (group C) received an equivalent volume of normal saline infusion. Post‐surgery, patient‐controlled intravenous analgesia (PCIA) was administered: group E received dexmedetomidine 0.5 mg/kg + alprazolam 50 mg + dexamethasone 5 mg, while group C received dexmedetomidine 0.5 mg/kg + dexamethasone 5 mg. Lactated Ringer's solution was used for goal‐directed fluid therapy (GDFT) during the procedure. Anesthesia induction, maintenance, and emergence were managed uniformly among groups. The protocol included intravenous midazolam 0.02 mg/kg, sufentanil 0.3 μg/kg, and etomidate 0.3–0.4 mg/kg. After loss of consciousness, rocuronium 0.2 mg/kg was administered intravenously. After 3 min of oxygenation and denitrogenation, a double‐lumen endotracheal tube was inserted under direct laryngoscopy, followed by mechanical ventilation. During surgery, remifentanil infusion rate and sevoflurane concentration were adjusted based on the patient's BIS values, hemodynamic indicators, and inhaled sevoflurane's minimum alveolar concentration (MAC) value. Intermittent intravenous rocuronium was given to maintain muscle relaxation.

In case of mean arterial pressure (MAP) < 65 mmHg or a decrease by 20% from baseline, intravenous epinephrine was administered for correction. If HR was < 50 beats/min, atropine 0.5 mg was administered. If MAP was < 65 mmHg (or decreased by 20% from baseline) along with HR < 50 beats/min, intravenous ephedrine 15 mg was administered.

### 2.3 Recording of Observational Parameters

General information of the subjects was recorded, including age, weight, surgical site, blood loss, fluid input, urine output, dosage of vasoactive drugs, etc. Continuous monitoring and recording were performed at the following time points: T_0_: upon entering the operating room; T_1_, before administration of alprazolam; T_2_, 10 min after alprazolam administration; T_3_, 15 min after alprazolam administration; T_4_, 20 min after alprazolam administration; T_5_, 5 min before surgery initiation; T_6_, start of surgery; T_7_, 5 min after surgery initiation; T_8_, 10 min after surgery initiation; T_9_, 15 min after surgery initiation; T_10_, end of surgery; T_11_, 5 min after the end of surgery; T_12_, 10 min after the end of surgery; T_13_, 1 day after surgery; T_14_, 3 days after surgery. The recorded parameters included SBP, DBP, MAP, and HR values.

### 2.4 Laboratory Indicators

**FIGURE 1:**
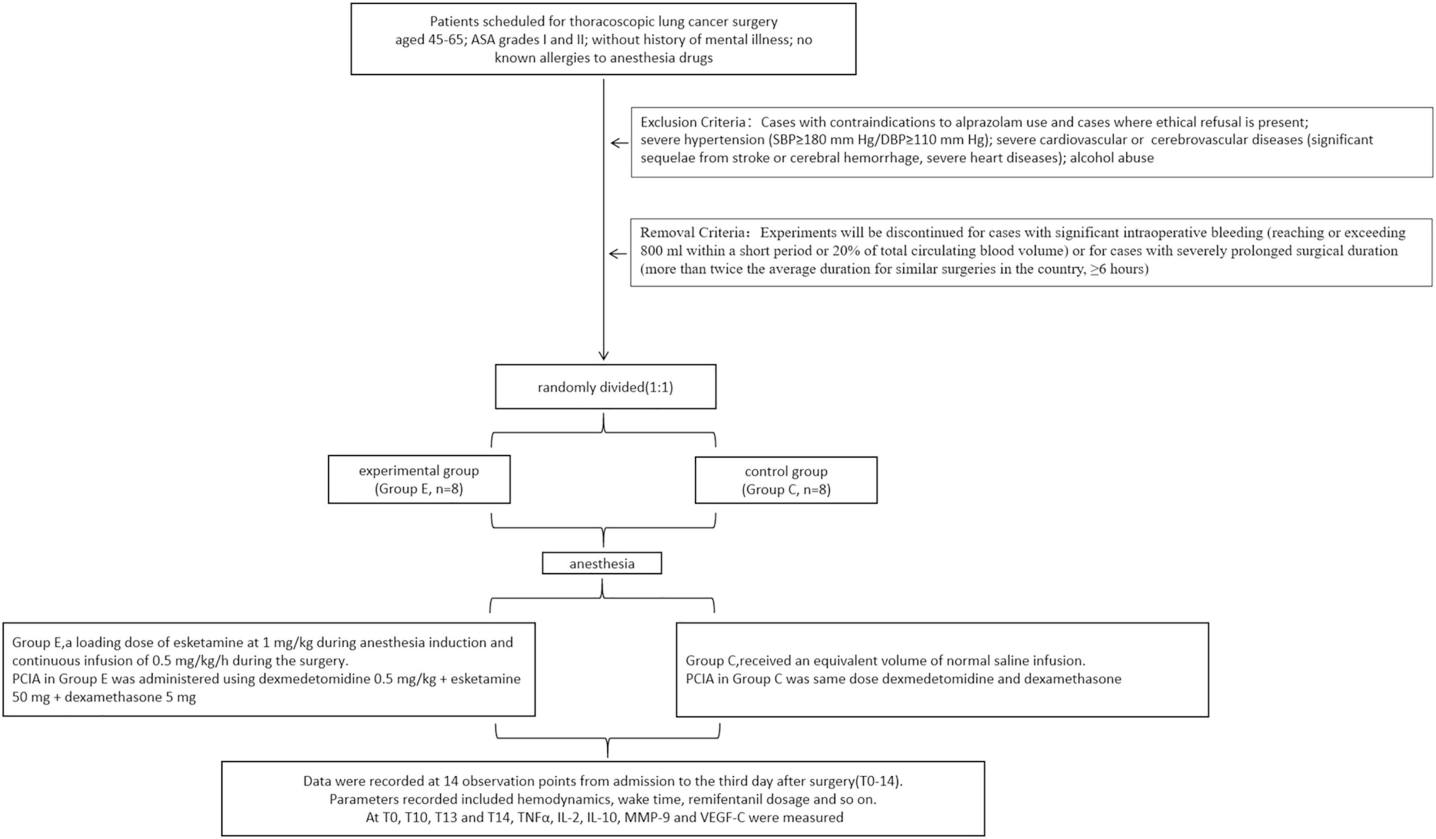
A CONSORT flowchart of the study design.

At time points T_0_, T_10_, T_13_, and T_14_, 4 mL of peripheral venous blood was collected from each subject and added to coagulation‐promoting tubes. After standing and centrifugation (3000 r/min, 20 min), the supernatant was transferred to a −60 °C freezer for storage. Concentration measurements of TNFα, IL‐2, IL‐10, MMP‐9, and VEGF‐C were conducted (Figure 1).

### 2.5 Statistical Analysis

Statistical analysis was conducted using SPSS 25.0 software. For continuous variables following a normal distribution, independent sample *t* tests were used, and the results were presented as mean ± standard deviation (x ± s). For continuous variables with skewed distribution, the Wilcoxon test was applied, and results were presented as median and quartiles. Chi‐square tests were used for categorical data, and a generalized estimating equation was employed for repeated measures comparisons of continuous data with subsequent simple effect analysis. *p* < 0.05 was considered statistically significant.

## 3 Results

### 3.1 Comparison of General Characteristics and Intraoperative Conditions

The general characteristics of age, weight, and surgical duration showed no statistically significant differences between the two groups (*p* > 0.05). In comparison with group C, group E exhibited prolonged emergence time (*p* < 0.05) (Table 1), and the remifentanil dosage in group E was lower than that in group C (*p* < 0.05) (Table 1).

**TABLE 1:** Comparison of general characteristics and intraoperative conditions between two groups of patients (*n* = 8).

	Group E	Group C	*p* value
Age (year, ^−^ *x* ± *s*)	57.1 ± 2.7	61.4 ± 2.6	0.278
Weight (kg, ^−^ *x* ± *s*)	67.9 ± 2.4	68.6 ± 2.6	0.847
Surgical site (numbers)			0.912
Upper lobe of left lung	2	2	
Lower lobe of left lung	1	2	
Upper lobe of right lung	2	2	
Middle lobe of right lung	0	0	
Lower lobe of right lung	3	2	
Awakening time (min, ^−^ *x* ± *s*)	24.3 ± 1.0	17.1 ± 0.4	0.001*
Operation time (min, ^−^ *x* ± *s*)	145.5 ± 9.1	150.9 ± 17.3	0.787
Total remifentanil (μg, ^−^ *x* ± *s*)	647.9 ± 106.9	1072.4 ± 325.6	0.007*
Blood loss (mL, ^−^ *x* ± *s*)	31.4 ± 7.5	31.9 ± 8.0	0.899
Fluid input (mL, ^−^ *x* ± *s*)	1668.8 ± 96.1	1681.2 ± 116.3	0.818
Urine output (mL, ^−^ *x* ± *s*)	121.2 ± 16.2	115.6 ± 19.5	0.945
Dosage of vasoactive drugs			
Norepinephrine (μg, ^−^ *x* ± *s*)	10.8 ± 2.4	8.2 ± 1.9	0.759
Atropine (mg, ^−^ *x* ± *s*)	0	0	0.999
Ephedrine (mg, ^−^ *x* ± *s*)	0	0	0.999

* There are significant differences between the two groups (*p* < 0.05).

### 3.2 Hemodynamic Indicators

**FIGURE 2:**
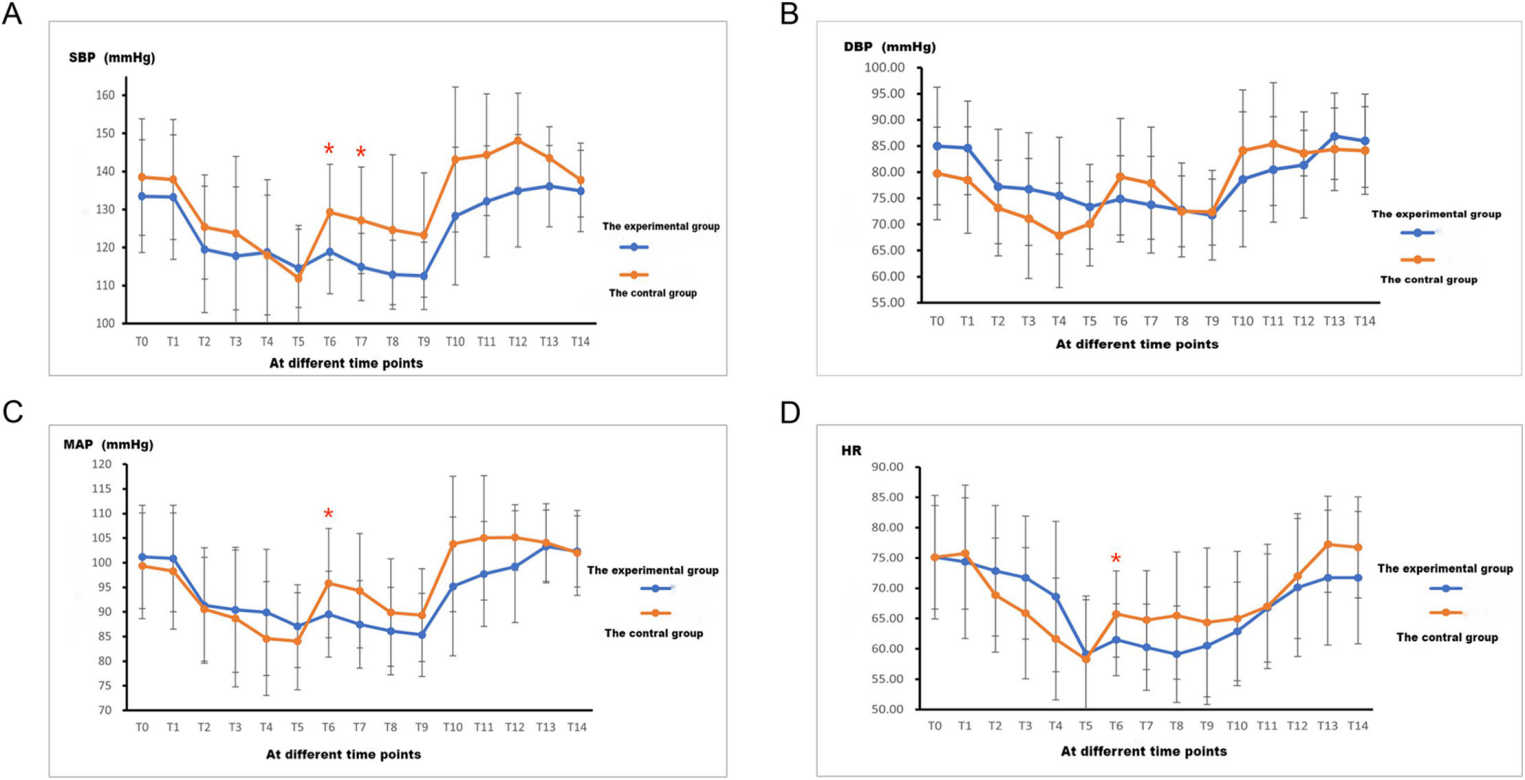
The SBP, DBP, MAP, and HR of the two groups at different time points.

Compared to T_0_, post‐induction SBP, DBP, MAP, and HR of the patients decreased, gradually increased after the start of surgery, and tended to stabilize. At T_6–7_, SBP in group E was lower than in group C (*p* < 0.05), with no statistically significant differences observed at other time points (*p* > 0.05, Figure 2A). There were no statistically significant differences in DBP comparisons at various time points (*p* > 0.05, Figure 2B). At the T_6_ time point, MAP and HR were lower in group E than in group C (*p* < 0.05), while there were no statistically significant differences between the two groups at other time points for MAP and HR (*p* > 0.05, Figure 2C,D).

### 3.3 Inflammatory Cytokines

#### 3.3.1 Comparison of Serum TNF‐α Concentration

**FIGURE 3:**
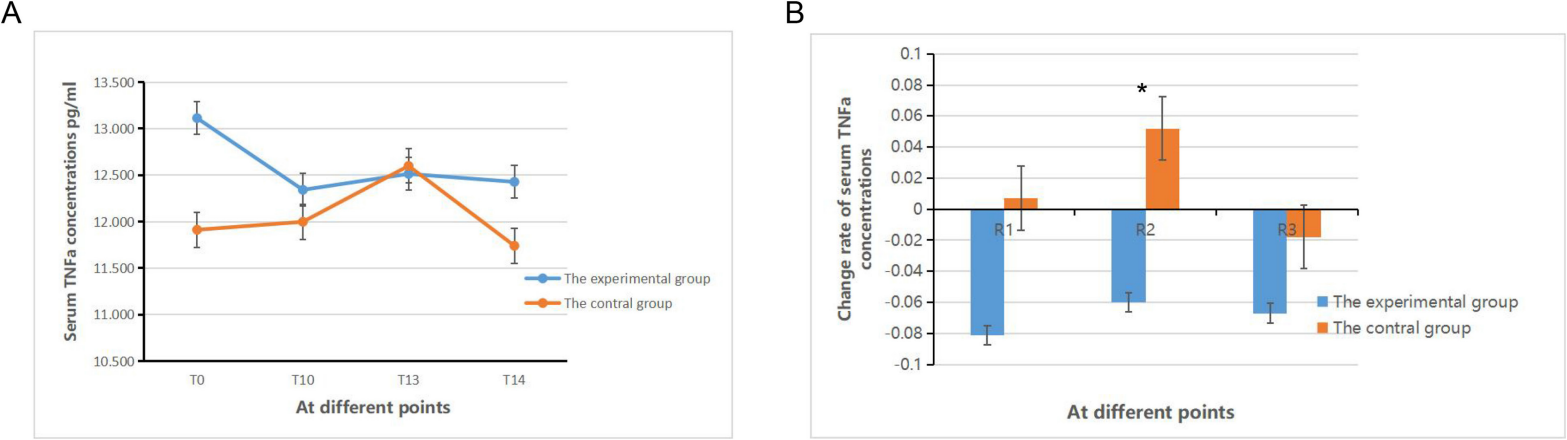
Change rate of serum TNF‐α concentrations in the two groups.

There was no statistically significant difference in the serum TNF‐α concentration between group E and group C at each time point (*p* > 0.05). However, there was a statistically significant difference in the serum TNF‐α concentration within each group at various time points (*p* < 0.05, Figure 3A). There was a statistically significant difference in R_2_ (*p* < 0.05) between group E and group C, while there were no statistically significant differences in R_1_ and R_3_ (*p* > 0.05). Within group E, there were no statistically significant differences in the comparisons of R_1_ to R_3_ (*p* > 0.05), whereas in group C, there were statistically significant differences in the comparisons of R_1_ to R_3_ (*p* < 0.05, Figure 3B).

#### 3.3.2 Comparison of Serum IL‐2 Concentration

**FIGURE 4:**
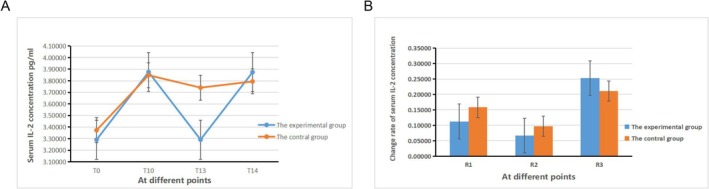
Change rate of serum IL‐2 concentration in two groups.

There was no statistically significant difference in the serum IL‐2 concentration between group E and group C at each time point (*p* > 0.05). However, there was a statistically significant difference in the serum IL‐2 concentration within each group at various time points (*p* < 0.05, Figure 4A). In group E and group C, there were no statistically significant differences in the comparisons of R_1_ to R_3_ both inter‐group and intra‐group (*p* > 0.05, Figure 4B).

#### 3.3.3 Comparison of Serum IL‐10 Concentration

**FIGURE 5:**
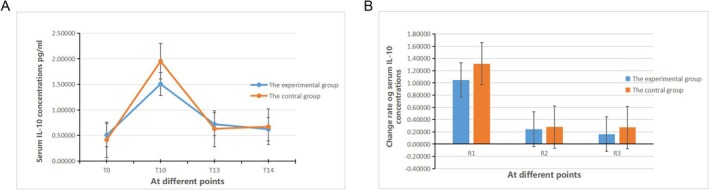
Change rate of serum IL‐10 concentrations in two groups.

There was no statistically significant difference in the serum IL‐10 concentration between group E and group C at each time point (*p* > 0.05, Figure 5A). Within group E, there were no statistically significant differences in the serum IL‐10 concentration at each time point (*p* > 0.05), while within group C, there were statistically significant differences in the IL‐10 serum concentration at each time point (*p* < 0.05). Between groups E and C, there were no statistically significant differences in the comparisons of R_1_ to R_3_ both inter‐group and intra‐group (*p* > 0.05, Figure 5B).

#### 3.3.4 Comparison of Serum MMP‐9 Concentration

**FIGURE 6:**
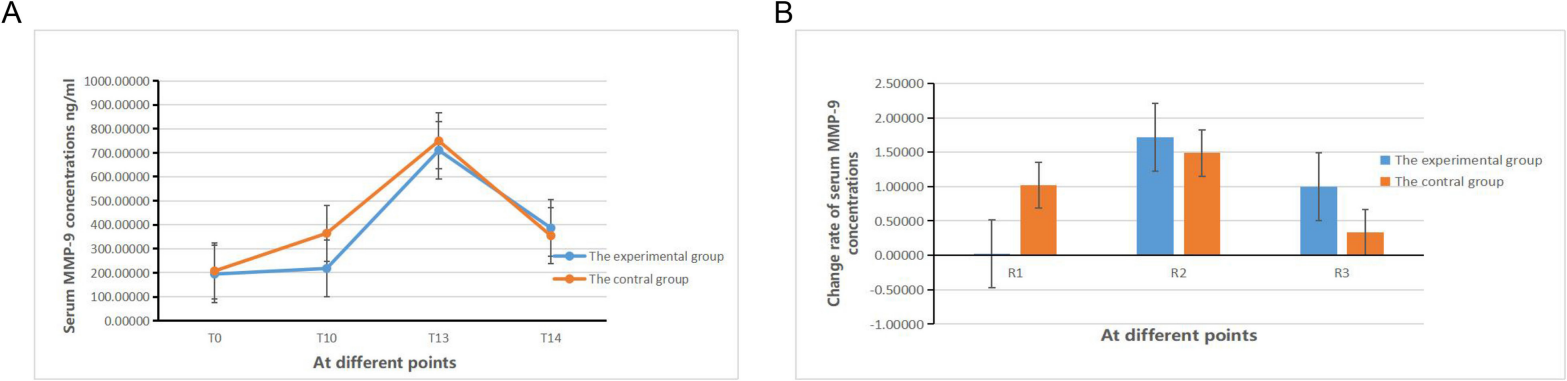
Change rate of serum MMP‐9 concentrations in two groups.

There was no statistically significant difference in the MMP‐9 serum concentration between group E and group C at each time point (*p* > 0.05). However, there were statistically significant differences in the MMP‐9 serum concentration within each group at various time points (*p* < 0.05, Figure 6A). Between groups E and C, there were no statistically significant differences in the comparisons of R_1_ to R_3_ inter‐group (*p* > 0.05). However, there were statistically significant differences in the comparisons of R_1_ to R_3_ within both groups (*p* < 0.05, Figure 6B).

#### 3.3.5 Comparison of Serum VEGF‐C Concentration

**FIGURE 7:**
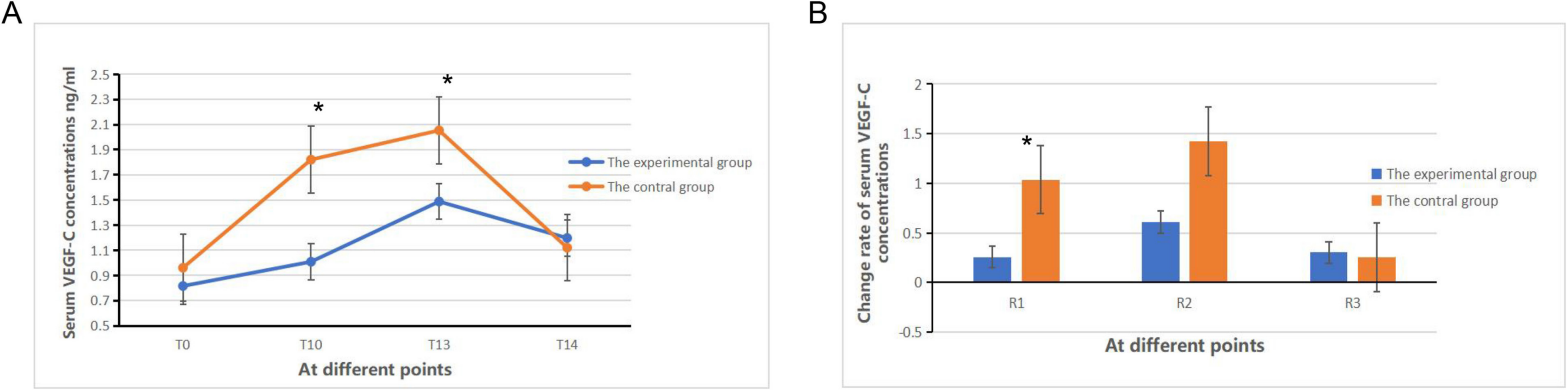
Change rate of serum VEGF‐C concentrations in two groups.

Between groups E and C, there was a statistically significant difference in the VEGF‐C serum concentration at the T_10_ and T_13_ time points (*p* < 0.05), while there were no statistically significant differences in the serum concentration at the T_0_ and T_14_ time points (*p* > 0.05). There were statistically significant differences in the VEGF‐C serum concentration within each group (*p* < 0.05, Figure 7A). Between groups E and C, there was a statistically significant difference in the comparison of R_1_ (*p* < 0.05), while there were no statistically significant differences in the comparisons of R_2_ and R_3_ (*p* > 0.05). There were statistically significant differences in the comparisons of R_1_ to R_3_ within both groups (*p* < 0.05, Figure 7B).

## 4 Discussion

Based on the pilot study, this research used the postoperative serum concentration of VEGF‐C as an indicator, with a mean of μ_1_ = 1.964 pg/mL for the control group and μ_2_ = 1.02 pg/mL for the experimental group. The standard deviation was σ = 0.40 pg/mL. With a significance level of α = 0.05 and a power of 1‐β = 0.8, the sample size was calculated using the formula,

$$n_{1}=n_{2}=\frac{2\left(Z_{\alpha /2}+Z_{\beta }\right)\sigma^{2}}{{\left(\mu_{1}-\mu_{2}\right)}^{2}}$$

The estimated minimum sample size was calculated to be *n* = 7; considering potential data loss, the sample size for each group was set at *n* = 8.

Esketamine is an NMDA receptor antagonist used as an induction agent in general anesthesia at doses of 0.5–1 mg/kg and for maintenance at doses of 0.5–3 mg/kg/h [23]. In this study, the induction dose was set at 1 mg/kg, and the maintenance dose was 0.5 mg/kg/h. Current research supports the sedative and analgesic effects of esketamine, which can reduce patients' stress response to surgical stimuli [18], aligning with the results of this study. Compared to group E, patients in group C exhibited a significant increase in SBP, MAP, and HR at the start of surgery. Moreover, the intraoperative remifentanil dosage in group E was significantly lower than in group C, indicating that esketamine enhances the sedative and analgesic effects of other anesthetics, thus reducing drug dosages. This is particularly relevant for cancer patients, where opioid sparing has practical protective implications.

The average emergence time for patients in group C was about 17 min, while it was approximately 24 min in group E, indicating a 7‐min prolongation. However, this difference does not hold substantial clinical significance for early postoperative complications, long‐term prognosis, or PACU management. Furthermore, after surgery, patients are typically transferred to the postanesthesia care unit (PACU), with such differences not affecting the efficiency of anesthesia and surgical procedures in clinical practice.

The video‐assisted thoracoscopic surgery can induce severe hemodynamic fluctuations and stress responses. This is manifested through sympathetic nervous system excitation, increased secretion of pituitary and adrenal cortex hormones [24], leading to heightened release of immune‐suppressive factors and pro‐inflammatory cytokines. In terms of inflammation, this is primarily driven by the upregulation of inflammatory mediators such as interleukins, TNF‐α, and MMPs, which activate relevant pathways and stimulate angiogenesis. Furthermore, the pre‐metastatic microenvironment establishes a pro‐angiogenic environment with high concentrations of VEGF and other pro‐angiogenic factors. This setting facilitates angiogenesis initiation and supports the metastatic progression of tumors. Experimental findings have indicated elevated serum concentrations of VEGF‐C in various tumor types compared to healthy individuals [13]. Hence, this study chose to assess serum concentrations of TNF‐α, IL‐2, IL‐10, MMP‐9, and VEGF‐C to reflect the conditions of the tumor microenvironment before metastasis. The objective was to observe the impact of perioperative esketamine administration on this environment.

Research has demonstrated that esketamine can alleviate inflammatory responses and reduce serum TNF‐α concentrations [25]. However, the results of this study indicated no significant differences in the TNF‐α serum concentrations at various time points between the two groups. Notably, there were differences in the change rate of TNF‐α serum concentration on the first day post‐surgery. This suggests that there were variations in TNF‐α levels between the two groups on the first day after surgery, with a downward trend in TNF‐α levels observed in group E and an upward trend in group C. TNF‐α, produced by macrophages and tumor cells, has dual roles in tumor development. Therefore, a comprehensive analysis of serum TNF‐α levels is necessary. In this study, patients in group E exhibited a decrease in serum concentration on the first day after surgery, whereas those in group C showed an increase. This could be attributed to esketamine inhibiting the immune response of patients, thus promoting tumor micro‐metastasis. Alternatively, it could be due to esketamine's capacity to suppress stress stimuli, alleviate inflammatory reactions, and thereby improve the tumor microenvironment, reduce the invasive capability of tumor cells, and inhibit tumor micro‐metastasis.

IL‐2 has a dual effect on immune responses; at high concentrations, IL‐2 promotes the proliferation of effector T cells, subsequently exerting anticancer activity, making it suitable for treating certain malignancies. However, at low doses, IL‐2 can inhibit immune activation and promote tumor cell metastasis. IL‐10 is a common anti‐inflammatory cytokine, but elevated levels of IL‐10 can suppress patient immune responses, thus promoting the formation of the tumor microenvironment before metastasis and increasing tumor cell immune evasion, ultimately facilitating tumor metastasis. The results of this study indicate that the IL‐2 and IL‐10 serum concentrations and their changes over time were consistent between groups E and C. However, previous research has demonstrated that sub‐anesthetic doses of ketamine can mitigate immune suppression and elevate IL‐2 levels [26], while esketamine can inhibit postoperative inflammatory responses and increase anti‐inflammatory cytokine IL‐10 levels. The discrepancies in research findings might be attributed to the multifactorial influence on IL‐2 and IL‐10 levels and various factors affecting patients' immune function and inflammatory responses during the perioperative period. Additionally, whether this effect is related to the enantiomer of ketamine warrants further investigation and differentiation.

The results of this experimental study indicate that there were no differences in the serum levels of MMP‐9 at various time points and the change rates relative to pre‐surgery levels between the two groups, suggesting that esketamine does not affect its expression. However, some research findings suggest that ketamine can reduce MMP‐9 synthesis, thereby inhibiting the formation of the tumor microenvironment before metastasis and restraining the proliferation and invasion of tumor cells [27]. Clinical trial results have shown that MMP‐9 serum concentrations are significantly elevated in malignant tumor patients [28]. Additionally, MMP‐9 can enhance the invasive and metastatic capabilities of tumor cells by degrading and remodeling the extracellular matrix and releasing vascular endothelial growth factors. This study indicates that esketamine does not affect the perioperative changes in MMP‐9 levels in lung cancer patients.

Patients in group E exhibited significantly lower postoperative VEGF‐C serum concentrations both immediately after surgery and on the first day compared to group C. Moreover, there was a significant difference in the change rate of VEGF‐C serum concentration between the two groups. VEGF‐C is a critical factor in the tumor microenvironment that promotes increased vascular permeability, migration of vascular endothelial cells, and neovascularization, thereby facilitating tumor cell metastasis. The results of this clinical study suggest that esketamine can reduce VEGF‐C release. However, the underlying mechanism remains unclear and requires further investigation.

Considering a comprehensive evaluation of the five indicators—inflammatory factors and circulating micro‐metastatic factors in the tumor microenvironment—esketamine appears to regulate the immune levels of patients undergoing video‐assisted thoracoscopic lung cancer resection. This regulation leads to a reduction in stress and inflammatory responses, which may contribute to improving the tumor microenvironment.

The limitation of this study is that it is a single‐center study with a small sample size and limited population diversity. In the future, we will strive to conduct further multi‐center studies with other regional hospitals to enrich the diversity of the population. The impact of esketamine on the tumor microenvironment was affirmed, but the mechanism and long‐term effects were limited to the experimental conditions and cannot be effectively observed. So, in‐depth study and long‐term observation can be conducted in the future.

## 5 Conclusion

The perioperative administration of esketamine in lung cancer patients offers evident sedative and analgesic effects, reducing the requirement for anesthetic drugs, alleviating surgical stress, and suppressing inflammatory responses. These effects may promote homeostasis within the tumor microenvironment.

## Author Contributions

Yong Wang performed the experiments and drafted the manuscript. Weijing Li and Li Jia collected and analyzed the data and prepared the figures. Junmei Shen and Chao Li supervised the algorithm development and analyzed the data. Huiqun Jia designed and supervised the study. All authors reviewed the manuscript.

## Ethics Statement

The experiments were approved by the Ethics Committee of The Forth Hospital of Hebei Medical University.

## Conflicts of Interest

The authors declare no conflicts of interest.

## Data Availability

The data in this study are available from the corresponding author upon request.
